# Global impact of COVID-19 pandemic on road traffic collisions

**DOI:** 10.1186/s13017-021-00395-8

**Published:** 2021-09-28

**Authors:** Yasin J. Yasin, Michal Grivna, Fikri M. Abu-Zidan

**Affiliations:** 1 Institute of Public Health, College of Medicine and Health Sciences, UAE University, Al-Ain, United Arab Emirates; 2 Department of Environmental Health and Behavioral Sciences, School of Public Health, College of Health Sciences, Mekelle University, Mekelle, Ethiopia; 3 Department of Surgery, College of Medicine and Health Sciences, UAE University, Al-Ain, United Arab Emirates

**Keywords:** COVID-19, Road traffic collision, Road safety, Injury, Death, Speed, Alcohol, Distraction

## Abstract

**Background:**

Various strategies to reduce the spread of COVID-19 including lockdown and stay-at-home order are expected to reduce road traffic characteristics and consequently road traffic collisions (RTCs). We aimed to review the effects of the COVID-19 pandemic on the incidence, patterns, and severity of the injury, management, and outcomes of RTCs and give recommendations on improving road safety during this pandemic.

**Methods:**

We conducted a narrative review on the effects of COVID-19 pandemic on RTCs published in English language using PubMed, Scopus, and Google Scholar with no date restriction. Google search engine and websites were also used to retrieve relevant published literature, including discussion papers, reports, and media news. Papers were critically read and data were summarized and combined.

**Results:**

Traffic volume dropped sharply during the COVID-19 pandemic which was associated with significant drop in RTCs globally and a reduction of road deaths in 32 out of 36 countries in April 2020 compared with April 2019, with a decrease of 50% or more in 12 countries, 25 to 49% in 14 countries, and by less than 25% in six countries. Similarly, there was a decrease in annual road death in 33 out of 42 countries in 2020 compared with 2019, with a reduction of 25% or more in 5 countries, 15–24% in 13 countries, and by less than 15% in 15 countries. In contrast, the opposite occurred in four and nine countries during the periods, respectively. There was also a drop in the number of admitted patients in trauma centers related to RTCs during both periods. This has been attributed to an increase in speeding, emptier traffic lanes, reduced law enforcement, not wearing seat belts, and alcohol and drug abuse.

**Conclusions:**

The COVID-19 pandemic has generally reduced the overall absolute numbers of RTCs, and their deaths and injuries despite the relative increase of severity of injury and death. The most important factors that affected the RTCs are decreased mobility with empty lines, reduced crowding, and increased speeding. Our findings serve as a baseline for injury prevention in the current and future pandemics.

## Introduction

The world is currently under the major impact of the COVID-19 pandemic. The disease spread swiftly and globally from Wuhan, China, the epicenter of the coronavirus, to the rest of the world, which was facilitated by fast transportation methods [1, 2]. Struggle is continuing to contain the spread of the repeated waves of infection. The dynamics of infectious pandemics is different from other natural disasters [3–5]. Although they do not cause mass destruction to the infrastructure, they significantly and directly affect the community health and economy [6]. The lack of strong evidence about the transmission routes of the virus at the beginning of the epidemic [1] and inadequate preventive measures increased the rapid spread of the virus [1, 3]. Overtime, better understanding of the infection routes with increased diagnostic tools, reporting, and tracing became more feasible [7–10]. Countries started implementing various national strategies to reduce the spread of the pandemic including physical distancing, quarantine, stay-at-home orders, closure of schools, restrictions of travel and mass gatherings, and complete lockdown [7, 8]. These measures reduced road traffic movements and changed traffic characteristics [3, 7], which in turn affected road traffic collisions (RTCs) [11]. Congestion, speed, density, and flow of traffic are interlinked [12]. The increased number of empty lanes following travel restrictions reduced road congestion and increased traffic flow [13, 14].

RTCs is a global health problem. It causes around 1.35 million deaths per year worldwide [15] costing around $1.8 trillion every year [16, 17]. In contrast, COVID-19 pandemic caused around 4 million deaths [18], and a global gross domestic product reduction of around 10% [19]. Understanding the impact of COVID-19 on RTCs is important and relevant to trauma surgeons and disaster medicine leaders. We aimed to review the effects of the COVID-19 pandemic on the incidence, patterns, and severity of the injury, management, and outcomes of RTCs and to give recommendations on improving road safety during this and future pandemics.

## Methods

### The planning phase

In the initial planning phase of this study, the authors discussed and aimed to review the different effects of the COVID-19 pandemic on RTCs. This was followed by structuring the general outline of different sections of the paper to be addressed. Based on this outline, we defined the search strategy, sources of literature, and inclusion and exclusion criteria for the literature search.

### Data source and search strategy

We followed several procedures to ensure a high-quality review. First, we searched electronic databases, mainly PubMed, Scopus, and Google Scholar, using a wide range of search terms and keywords or a combination of keywords on the two central concepts: COVID-19 and RTC, to retrieve the published articles. The keywords include COVID-19, SARS-CoV-2, novel, coronavirus, pandemic, lockdown, quarantine, stay-at-home, shelter-in-place, safer-at-home, social restriction, social distancing, road, traffic collisions, traffic accidents, traffic crashes, motor vehicle, fatality, mortality, death, injury, travel behavior, mobility behavior, public transport, speed, drug, alcohol use, drinking, driving, psychological stress, anxiety, depression, trauma centers, emergency, orthopedic, trauma, emergency surgery, and trauma volume. Second, bibliographic references of these retrieved articles were used to obtain additional relevant papers. Third, the google search engine was used for collecting published discussion papers, reports, and media news. Finally, as a fourth step, several websites were searched to extract and collect published literature.

### Inclusion and exclusion criteria

We included peer-reviewed journal articles, discussion papers, reports, and media news related to the effect of the COVID-19 pandemic on RTCs over the last 18 months, which is up to June 2021. No exclusions were made based on the article types, methodology of the study, outcome reported, study period, the scope of the study, and study setting. In other words, any literature related to the effect of the COVID-19 pandemic on RTCs was included. Literature was excluded if the language of publication was not English.

### Data extraction and synthesis

Based on the primary outline of the study, the findings were critically summarized, data extracted, combined and synthesized using a narrative synthesis.

## Results and discussion

### Effects on traffic congestion

**Fig. 1:**
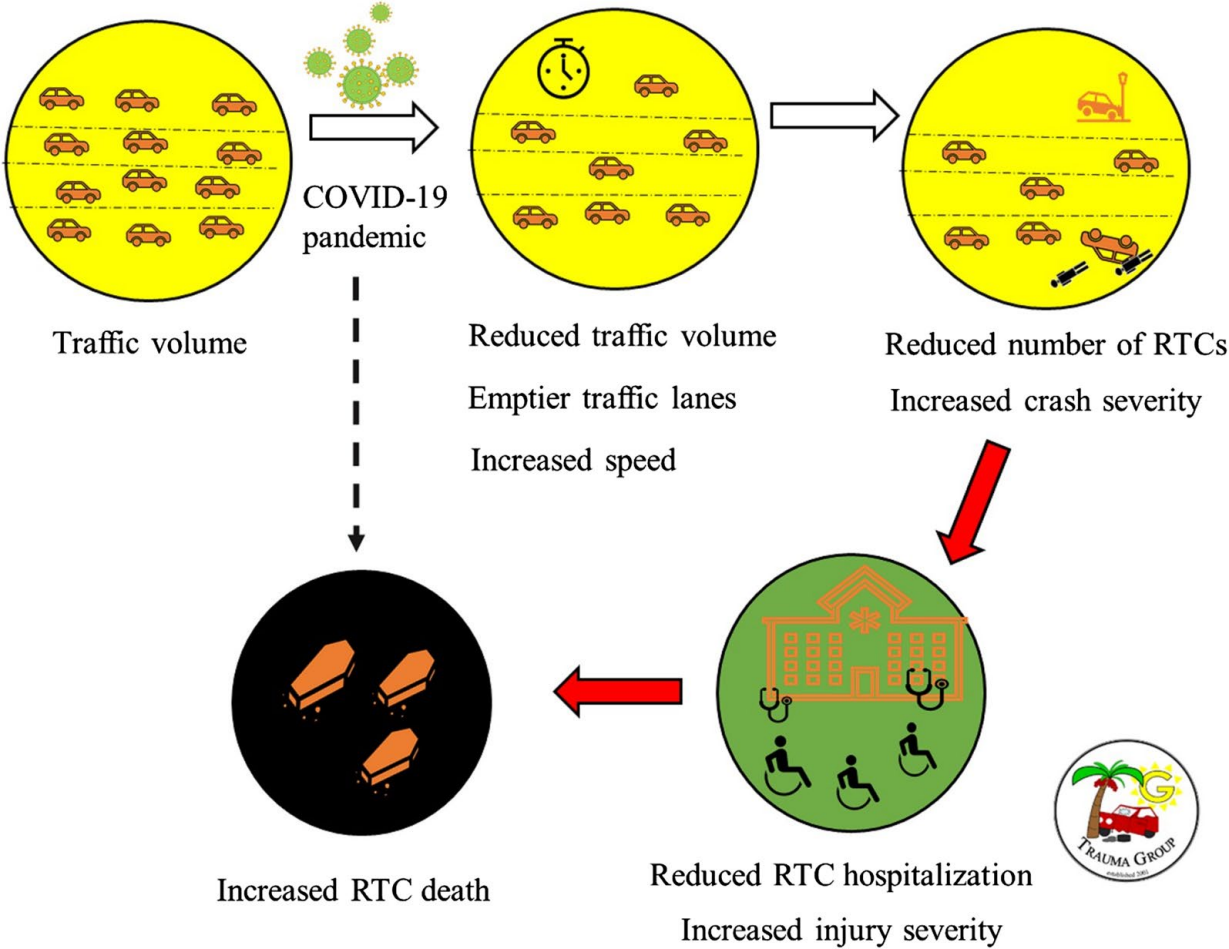
Overall impact of the COVID-19 pandemic on traffic volume, traffic lanes, vehicle speed, number of road traffic collisions, injury severity, hospitalization, and RTC deaths

Reduction in traffic congestion affects speed, traffic flow, and traffic density [12, 20]. Reduced mobility may decrease vehicle congestion, which in turn reduces road traffic collisions [21–26]. In contrast, it will increase empty road lanes, which may increase speeding (Fig. 1). During the COVID-19 pandemic, travel restrictions had significantly reduced vehicle mobility [27–32] which was reduced by more than 50% worldwide [28], with a decrease of 50 to 60% in the Asian countries [28] and 55 to 80% in the European countries [11, 22, 33, 34]. These resulted in a significant decline in public transport (60 to 80%), private cars, driving, and walking globally during March–May 2020 [28–30, 35–37]. Furthermore, there was a significant reduction among public transport users in Latin America and the Caribbean (by 60 to 90%) [28, 38] and European countries (by 40 to 90%) [21, 22, 30, 33, 39]. Similarly, there was a reduction in car trips (or private car trips) by 65 to 80% in European countries [33, 40]. Overall, these resulted in a sharp decrease in traffic congestion [41, 42].

The reduction of traffic congestion has varied considerably in different countries [41, 43, 44]. It ranged between 25 and 75% during lockdown periods in different European countries, with the highest decrease of 75% in France, Spain, and Italy in April 2020 compared with April 2019 [43] (Table 1). Similarly, traffic reduced by 40% in the USA [45], 63% in the UK [46], 77% in South Africa, 74% in New Zealand, 60% in Israel, 59% in Mexico, and 43% in Australia during the same period compared with April 2019 [43]. It is worth noting that the changes in traffic reduction may fluctuate depending on the comparison periods [43, 44] (Table 1). Traffic reduction varied by the local setting, jurisdictions [23, 47–51], type and function of the road [43, 44], residence (urban and rural) [41, 44, 52] and vehicle types [43, 44, 46].

**Table 1:** Road traffic and road deaths during COVID-19 pandemic

Country	Traffic volume (% change)	Road deaths (% change)		Annual road deaths (% change)	References
	April 2020 versus April 2019	April 2020 versus April 2019	April 2020 versus April 2017–2019 average	2020 versus 2019	
Albania	NA	NA	NA	− 20	[117]
Australia	− 43	− 23 to − 24	NA	− 7	[43, 135, 136]
Austria	− 50	− 25 to − 30	− 9	− 19	[24, 43, 44]
Belgium	− 60 *March 2020 versus March 2018–2019	− 68	− 68	− 22	[24, 44]
Bosnia and Herzegovina	NA	NA	NA	− 7	[117]
Bulgaria	NA	NA	NA	− 26	[24]
Canada	NA	− 34	− 46	+ 22 *Ontario	[43, 101, 137]
Chile	− 56.5 (Santiago city)	− 24 (June on June)	NA	NA	[43]
Columbia (*Cali)	− 70 *April 2020 versus 2016–2019		− 55 *Compared to 2016–2019	NA	[138]
Croatia	− 50 *April 2020 versus April 2018–2019	− 39	− 35	− 20	[24, 44]
Cyprus	− 65 (urban areas) and − 70 (motorways) *April 2020 versus February 2020	− 100	− 100	− 8	[24, 44]
Czech Republic	− 50 (motorways)	− 11 to − 15	+ 5	− 16	[24, 43, 44]
	− 65 (urban areas) *April versus February 2020 and before				
Denmark	− 25	+ 9	+ 6	− 22	[24, 43, 44]]
Estonia	− 10 *April 2020 versus April 2018–2019	− 25	− 18	+ 15	[24, 44]
Finland	− 34	− 24 to − 29	− 38	+ 4	[24, 43, 44]
France	− 75	− 56	− 61	− 21	[24, 43, 44]
Germany	− 48	− 1	− 5	− 11	[24, 43, 44]
Greece	− 42 to − 74 *March–April 2020 versus Feb 2020	− 58	− 59	− 16	[24, 43, 44, 70]
Hungary	− 33	− 43	− 49	− 25	[24, 43, 44]
	− 41 *April 2020 versus April 2017				
Iceland	NA	NA	NA	+ 33	[24]
Ireland	− 62 (Cars)	− 22	− 36	+ 6	[24, 43, 44]
	− 65 to − 70 (National road network)				
Israel	− 60	− 28	NA	NA	[43]
Italy	− 75 to − 80	− 79 to − 80	− 84	− 25	[24, 43, 44]
Japan	NA	NA	NA	− 12	[119]
Kosovo	NA	NA	NA	− 28	[117]
Latvia	NA	− 33	− 25	+ 7	[24, 44]
Lithuania	− 36	− 42 to − 71	− 30	− 6	[24, 43, 44]
Luxembourg	NA	NA	+ 300	+ 18	[24, 44]
Macedonia (North)	NA	NA	NA	− 5	[117]
Malta	NA	NA	NA	− 31	[24]
Montenegro	NA	NA	NA	+ 2	[117]
Mexico	− 59	− 23	NA	NA	[43]
Morocco	NA	− 65	NA	NA	[43]
Nepal	− 59 *March 24–June 14, 2020 versus 2019	NA	NA	NA	[57]
Netherlands	− 35	+ 6	+ 13	− 8	[24, 43, 44]
New Zealand	− 74	− 80	NA	− 9	[43, 139]
Norway	− 25	− 46	− 19	− 11	[24, 43, 44]
Poland	NA	− 32	NA	− 15	[24, 43]
Portugal	NA	− 50 to − 59	− 46	− 18	[24, 43, 44]
Romania	NA	− 46	− 52	− 12	[24, 44]
Saudi Arabia	− 26 to − 55 *March–April 2020 versus February 2020	NA	NA	− 20	[70, 118]
Serbia	NA	− 49	NA	− 8	[43, 117]
Slovakia	NA	+ 50	+ 20	− 9	[24, 44]
Slovenia	− 54	− 42	− 40	− 22	[24, 43, 44]
South Africa	− 77	− 78	NA	NA	[43]
South Korea	NA	NA	NA	− 12 *Seoul	[140]
Spain	− 75 (− 75 *April 2020 versus April 2017–2019)	− 49 to − 59	− 63	− 21	[24, 43, 44]
Sweden	− 22 (− 22 *April 2020 versus Apr 2017–2019)	+ 6	+ 2	− 14	[24, 43, 44]
Switzerland	NA	NA	NA	+ 21	[24, 124]
Uruguay	NA	− 51	NA	NA	[43]
UK	− 63	− 48	NA	− 14	[46, 109]
USA	− 40	− 18	NA	+ 7	[45, 110]

*NA* not available

The drop in traffic congestion was not only related to the COVID-19 lockdown. Traffic declined in Sweden (by 22%) and Netherland (by 35%), although they were not under lockdown [43, 44]. Reduced transportation has negative effects on the economy [19, 43]. Travel by private cars increased when restrictions were lifted [53]. In India, more than 90% of public transport users felt unsafe to be infected compared with 13% of those using private transportation [54]. Traffic congestion of major cities was reduced by around 15% worldwide. Nevertheless, it increased by 4% in few cities like Changchun in China [41]. Analyzing traffic congestion indicates that vehicle travel distance is one of the risk factors for RTCs [55].

### Effects on vehicle speed

Excessive speed, which is anticipated to increase the incidence and severity of RTCs [12], has occurred during the COVID-19 pandemic [20, 56–61]. This was attributed to the significant reduction in traffic volumes and empty roads [42, 44, 58, 59, 62, 63] which encouraged high speeding [20, 42, 47, 56, 57, 60, 64], and resulted in more RTCs despite the few numbers of vehicles [56, 60]. Furthermore, traffic speed enforcement was less [20, 44]. Over-speeding increased by 39% in Spain, 22% in Estonia, 16% in France, and 10% in Denmark [44]. Extreme speeding offenses increased by 236% in the UK [63]. In the USA, over-speeding increased in various metropolitan cities by 13 to 64% during the COVID-19 restrictions [56].

High speed was a key factor for the rise in road fatalities in Northern Ireland and USA despite the quarantine [20, 48, 51, 62]. Road fatalities, attributed to over-speeding and unbelted passengers, increased by 78% in Virginia, USA, despite the reduced incidence of road collisions by 45% [65]. These results were inconsistent in all countries. Although serious speeding increased in France and Germany, road collisions in these countries decreased by 74% and 23%, respectively [44]. In contrast, the speed-related vehicle collisions decreased in Minnesota (USA) and the Czech Republic despite reduced traffic volumes during lockdown [23, 44]. In Nepal, 25% of the RTCs were attributed to speeding during the pandemic [57].

### Effects on traffic lanes

The number of empty/open traffic lanes increased during the COVID-19 pandemic, which was attributed to the reduced traffic volumes following travel restrictions [44, 62, 66, 67]. The impact of these empty/open traffic lanes on excessive speed and associated accidents has been less studied. A report by the National Safety Council, USA, showed that the increased number of empty traffic lanes contributed to excessive speed and increased road death rates in several states during the quarantine [62]. Another study found that empty lanes were associated with increased speed and collisions [14].

Furthermore, several major cities across the globe, such as Philadelphia, Calgary, Berlin, Bogota temporarily replaced traffic lanes with sidewalks and bike lanes to support walking and bicycling by providing more space for pedestrians and cyclists [44, 66]. This was an attempt to improve physical activity and mitigate the psychological effects of the COVID-19 pandemic [6]. Empty lanes encouraged cycling in different major cities of Europe [67]. On the contrary, these empty lanes encouraged extreme speed that resulted in fatality crashes in several other cities [63].

### Effects on driving behaviors

Understanding the driving behavior of traffic users is essential for improving road safety during the pandemic [68, 69]. A study from Greece and Saudi Arabia found that the reduced traffic volume during the lockdown increased speed, harsh acceleration, repeated harsh braking, and the usage of mobile cellphones [70]. Adolescent driving time and distance decreased by around 35%, during movement restriction. These changes were less in older, employed, and ethnic minority adolescents but greater in those with greater social tendencies [71]. Interestingly, there was generally a reduction in distraction-related RTCs in Louisiana, USA by 43% during the lockdown, although there was a slight increase in injuries among drivers using mobile phones [72]. Similarly, fatalities with unrestrained drivers increased by 15% in Virginia [65] and 11% in Minnesota, USA [73] indicating a decrease in seat belt compliance [20, 74]. During the COVID-19 pandemic drivers in US and Canada were more likely to drive distracted, not wearing seat belts, over-speeding, and using drugs [75, 76].

### Effects on drug and alcohol abuse

The impact of the COVID-19 pandemic on drug and alcohol-induced traffic collisions has been less studied compared with the impact of the pandemic on health behavior changes. Travel and social restrictions, psychological stress, anxiety, and fear of exposure to the virus were associated with increased alcohol consumption during the pandemic [77–80]. Alcohol consumption was increased by 14% in Washington, USA [77]. Increased alcohol and drug consumption were attributed to more free time during the COVID-19 pandemic [61, 78]. This may lead to over-speeding, impaired driving, and stunt driving, with reduced road safety [20, 61, 72, 81]. Increased drug and alcohol consumption may lead to increased suicide, domestic violence [82], and risky driving [20, 72, 75, 81–83]. Thomas et al. found a significantly higher overall prevalence of drug and alcohol use in seriously and fatally injured RTCs patients, being 65% during the pandemic compared with 51% before that [81]. This included an increase in alcohol use, cannabinoids, and opioids (28% compared with 22%, 33% compared with 21%, and 14% compared with 8%, respectively) [81]. However, other studies from Canada and USA showed a slight reduction (around 3%) in alcohol use during the pandemic [75, 83].

### Effects of psychological impact on RTCs

The COVID-19 pandemic has a major psychological impact on the community affecting the mental health and vulnerable groups. This is exaggerated by the preventive measures taken to fight the virus, including quarantine, lockdown, self-isolation, and social distancing, which are unusual human behaviors [6, 84, 85]. Negative emotions, such as depression and anxiety, increased after the COVID-19 outbreak [86–88]. Fear of dying, death of family members, fear of stigma, reduced social interaction, and lockdown are potential risk factors of mental health problems [6]. Furthermore, the global failure to find an effective treatment or vaccine for the pandemic, or their shortages is another factor causing psychological pressure, not only to the infected persons but also to the healthy individuals [89]. Negative emotions such as depression, sadness, anxiety, stress, fatigue, distraction have a deleterious effect on driving behaviors and speeding [90–92]. The effects of these negative emotions on driver performance and speeding during the COVID-19 pandemic and its impact on RTCs have not been adequately examined [93]. COVID1-19 pandemic may negatively affect drivers’ mood, which is associated with anxiety and aggressive driving leading to tragic outcomes [94].

### Incidence of RTCs

RTCs are related to the risk of exposure to traffic, human behavior, vehicle design, traffic volumes, road infrastructure, and environmental conditions [12, 23, 95, 96]. Although travel restrictions have different effects on the above factors, the effects on road infrastructure, environment, and vehicle design are unchanged. Traffic volume and open/empty traffic lanes possibly affect human behavior [12, 13, 26, 97]. Road traffic collisions during the COVID-19 restrictions were generally reduced globally [11, 21, 44, 51, 56, 98–100] which is attributed to the reduced traffic volumes [21, 23, 40, 44, 47]. This reduction varies considerably by country and the type and function of roadways [56]. It was 67% in Spain [11], 26% in Canada [101], 84% in UAE [102], 48% in Nepal [57], 30–60% in Turkey [99, 103], 11 to 58% in USA [23, 56, 59, 72, 104], 29 to 53% in Northern Ireland [51, 105], 74% in France, 28% in Czech Republic, and 23% in Germany [44] during lockdowns.

### Severity of RTCs

It is important to clarify that although the numbers of RTCs may have reduced and the absolute number of RTC death may decrease, the relative percentage of victims having severe injuries or death may increase despite the overall reduced standardized RTC population death rate [42, 56, 59, 98], (Fig. 1). For example, the decline of RTCs in Missouri, USA, during the mandated lockdown resulted in decreased mild injuries, but not in serious/fatal injuries [98].

This can be attributed to the increased speed [14, 59, 106], empty lanes, and reduced law enforcement during COVID-19 lockdown [20, 42, 44, 62]. Severe injuries will increase the morbidity, mortality of injured patients, and medical treatment costs [59, 107]. Speeding was the main factor for fatal collisions during the COVID-19 lockdown [42, 56, 59]. The ratio of deadly crashes to all crashes dramatically increased due to excessive speed by 470% in Madrid (Spain), by 292%, in Chicago, by 167% in New York, and by 65% in Boston [63]. The fatality rate increased by 14% per miles driven across the US states during March 2020 [62] and by 37% per miles driven during April 2020 [63], which was attributed to extreme speed [42, 56]. This was reflected in studies from trauma centers that demonstrated an increase of injury severity of an ISS above nine of admitted patients from 35% before the lockdown to about 63% during the lockdown [108].

As explained above, the absolute number of fatal accidents, severe injuries, and mild injuries of RTCs declined by 41%, 8%, and 42%, in Greece, respectively [70]. Similarly, in Australia, the absolute number of fatal crashes decreased by 10% in 2020 compared with the prior three-year, which varied depending on jurisdictions, except in Queensland, where it increased by 11% [49]. Likewise, there was a reduction in the number of fatal collisions by 35% in New York City and 56% across mainland France in April 2020 compared with April 2019 [63].

### Outcome of RTCs

The significant reduction of RTCs during the COVID-19 lockdown had different clinical outcomes [43, 44]. There was a significant decrease in the number of traffic-related deaths and injuries in most of the countries. Nevertheless, the opposite occurred in others. This is because the decline in traffic may increase risky driving behaviors such as over-speeding with increased severity of injury despite the decrease in the incidence of RTCs [43, 56, 59, 75, 81]. The strict lockdown during the pandemic reduced the number of RTCs deaths globally [43, 44, 57, 99, 109–111] (Table 1).

In contrast, the absolute number of road deaths in April 2020 increased in Slovakia (50%) and Denmark (9%) compared with April 2019 [43, 44]. Similarly, there was an increase in the number of road fatalities in The Netherland (6%) and Sweden (6%) in April 2020 compared with April 2019, although there were no lockdown measures in these countries [43, 44].

Death is mainly caused by high-energy transfer from the vehicle to the road traffic users during the crash. The mortality of pedestrians and motorcyclists are much higher compared with vehicle occupants in the same setting because the pedestrians and motorcyclists are vulnerable road users (pedestrians, cyclists, and motorcyclists) compared with the vehicle occupants [112, 113]. The interaction between the road infrastructure, environment, size and speed of a vehicle, behavior of drivers, traffic mobility, congestion, empty lines will explain the differences in mortality between different states and countries [114–116]. The reduction of road deaths on rural roads in Spain during lockdown by 62% resulted in a 10% reduction of the number of road deaths among vulnerable road users (from 37% before lockdown to 27% during lockdown) [44]. Similarly, the reduction of death or seriously injured pedestrians (by 24%) and motorcyclists (by 16%) were less compared with passengers (by 38%) in Northern Ireland, UK [51]. In Australia, there was reduction of the number road traffic deaths among pedestrians (by 20%), motorcyclists (by 12%), passengers (by 11%), and drivers (by 5%), but cyclists death increased by 29% during the initial stage of the lockdown compared with the prior three years [49]. In contrast, road fatality among vulnerable road users increased in the Czech Republic during the lockdown (by 27%); cyclists and motorcyclists death increased by 86% and 50%, respectively [44].

Notably, the effect of the COVID-19 pandemic on road deaths continued after the lockdown periods. Overall, the number of annual road death dropped significantly in most countries. However, few had the opposite. There was an overall reduction in the annual absolute number of road deaths in most of the 27 European countries by 17% [24], six Balkan countries by 11% [117], in Saudi Arabia by 20% [118], and in Japan by 12% [119, 120]. In contrast, the absolute number of road deaths increased in some countries like Luxembourg by18%, Ireland by 6%, Finland by 4%, and Switzerland by 21% [24, 117]. Similarly, it generally increased in the USA by 7% [110] with major variations in different states [121].

### Hospitalization in trauma centers

The reduction of RTCs during the COVID-19 pandemic reduced the number of treated trauma patients at trauma centers globally by 20 to 85% compared with previous years [25, 60, 107, 108, 122–127]. This was shown in different countries worldwide like China [126], Spain [125], India [127], South Africa [25], UK [60, 105, 108], USA [107, 128–130], Australia [124], Ireland [131], and New Zealand [132]. A study conducted by Ajayi et al. (2020) in UK found a decrease in trauma admissions during COVID-19 lockdown despite an increased incidence of road collisions [133] (Table 2).

**Table 2:** Impact of COVID-19 pandemic on the burden of trauma centers and percentage change of RTC

Country	Trauma center	Trauma admission (% change)	RTC (% change)	Findings/interpretation	References
		2020 versus 2019 or previous years	2020 versus 2019		
Australia	Westmead Hospital Trauma Registry	− 23 to − 34 *2020 versus 2016–2019	− 40 to − 52 *2020 versus 2016–2019	Overall, trauma admission reduced by 23–34%, and number of RTC admission reduced by 40–52%	[124]
India	Tertiary care referral hospital, North India	− 34 to − 73	− 51 to − 86	Overall, trauma admission reduced by 34–73%, And number of RTC admission reduced by 51–86%	[127]
	Multi-centre	− 63	80	Overall, trauma admission was reduced by 63%, and number of RTC admission reduced by 80%	[141]
Ireland	Connolly Hospital, Dublin, Ireland	− 40	− 60	Overall, trauma admission was reduced by 40%. Number of RTC admission reduced by 60%	[131]
New Zealand	Midland Trauma Registry	− 43 *March 5–18. 2020 versus March 26–April 8, 2020	− 21 *March 5–18. 2020 versus March 26–April 8, 2020	Overall, trauma admission was reduced by 43%. Number of RTC admission reduced by 21%	[132]
South Africa	Edendale Hospital Emergency Department, in Kwa-Zulu Natal	− 47 *April–May 2020 versus February–March 2020	− 78	Overall, trauma admission was reduced by 47%. Number of RTC admission reduced by 73%	[25]
	Groote Schuur Hospital (GSH), Cape Metro West Health District, Cape Town	− 53	− 74	Overall, trauma admission was reduced by 53%. Number of RTC admission reduced by 74%	[142]
UK	Royal Victoria Hospital (RVH) Belfast, Northern Ireland	− 26	− 53	Overall, trauma admission was reduced by 26%. Number of RTC admission reduced by 53%	[105]
	Aintree University Hospital, Liverpool, England	− 38	− 43	Overall, trauma admission was reduced by 38%. Number of RTC presentation reduced by 43%	[108]
	North-West London Major Trauma Centre	− 46	− 48	Overall, trauma admission was reduced by 46%. Number of RTC presentation reduced by 48%	[60]
USA	Les Angeles County trauma center	− 2	NA	Trauma decreased with fewer motor vehicle collisions	[128]
	Multicenter study of US trauma centers	− 11	− 27	Overall, trauma admission was reduced by 11%. There was a decrease in number of RTC admission by 27%	[129]
	UC Davis Medical Center	NA	− 38	38% reduction in motor-vehicle injury	[47]
	McLaren Oakland Hospital (MOH) in Pontiac, MI	− 45 *2020 versus March–April 2016–2019	− 46	Overall, trauma admission reduced by 45%. There was a decrease in RTC admission by − 46%	[107]
	Portsmouth Regional Hospital	− 57 *February–April 2020 versus 2017–2019	− 81 *February–April 2020 versus 2017–2019	Overall, trauma admission was reduced by − 57%. RTC presentation reduced by 81%	[130]

*NA* not available

### Cost

A significant amount of money was saved from avoiding collisions during the COVID-19 pandemic. These savings are related to material damage, human cost (life and pain), treatment of injuries, lost time at work, and administrative costs, including insurance claims and emergency response [12, 47]. California State, USA, saved more than one billion USD due to reductions in vehicle collisions after the lockdown order [47]. The savings ranged from 7 to 24 billion USD in other five US states [104]. Despite that, the costs of RTCs are still expensive despite the quarantine [134]. Overall, the cost of motor vehicle deaths, injuries, and property damage in the USA was estimated to be more than 474 billion USD in 2020 [121].

### Recommendations

- Ensuring road safety legislations, including its enforcement, driving license requirements, and alcohol consumption regulations during the COVID-19 pandemic.
- Collaborative efforts among different stakeholders, including research institutions, police, hospitals, transport and safety departments, insurance companies on national and international levels to coordinate and act on the needed response to increase road safety during infectious pandemics.
- Review the lessons learned on RTCs during the COVID-19 pandemic and use them to mitigate the effects of the current and future pandemics.
- Have a comprehensive digitalized data record to study the impact of the COVID-19 pandemic on road safety and use the generated data to monitor and plan future disaster management of pandemics.
- Carry out comprehensive studies on road safety during the COVID-19 pandemic with appropriate methodological approach and research funds. This should include studies from rural areas and developing countries.

## Conclusions

The COVID-19 pandemic has generally reduced the overall absolute numbers of RTCs, and their deaths and injuries despite the relative increase of severity of injury and death. The most important factors that affected the RTCs are decreased mobility with empty lines, reduced crowding, and increased speeding. Our findings serve as a baseline for injury prevention in the current and future pandemics. Future research with a comprehensive methodological approach that includes rural areas and developing countries is demanded.

## Data Availability

Not applicable.

## Abbreviations

RTC: Road traffic collisions
UAE: United Arab Emirates
UK: United Kingdom
USA: United States of America
USD: United States Dollar
